# *Triatoma sanguisuga* Blood Meals and Potential for Chagas Disease, Louisiana, USA

**DOI:** 10.3201/eid2012.131576

**Published:** 2014-12

**Authors:** Etienne Waleckx, Julianne Suarez, Bethany Richards, Patricia L. Dorn

**Affiliations:** Loyola University New Orleans, New Orleans, Louisiana, USA

**Keywords:** *Triatoma sanguisuga*, Chagas disease, blood meals, 12S rDNA, *Trypanosoma cruzi*, parasites, Louisiana, vector-borne infections

## Abstract

To evaluate human risk for Chagas disease, we molecularly identified blood meal sources and prevalence of *Trypanosoma cruzi* infection among 49 *Triatoma sanguisuga* kissing bugs in Louisiana, USA. Humans accounted for the second most frequent blood source. Of the bugs that fed on humans, ≈40% were infected with *T. cruzi*, revealing transmission potential.

Chagas disease, caused by the parasite *Trypanosoma cruzi*, is mainly transmitted to humans and other mammals by blood-sucking insects called triatomines (also known as kissing bugs). In the United States, 24 species of wild mammals have been found to be naturally infected with *T. cruzi*, but only a few (<25) autochthonous cases of vectorial transmission to humans have been described (*1*,*2*). This number is probably an underestimate, and there is concern that vectorial transmission to humans in the United States may increase because of the following factors: 1) loss of sylvan blood sources because of habitat destruction, forcing the bugs to seek other (possibly human) blood sources; 2) climate change that could extend the range of the vectors northward; and 3) introduction of parasites by migrants from disease-endemic countries (*3*–*5*). Among the 11 triatomine species in the United States, the most widely distributed and the only 2 found in Louisiana are *Triatoma lecticularia* and *T. sanguisuga* (*5*). Bugs of the species *T. sanguisuga* are responsible for the first described autochthonous case of *T. cruzi* transmission in Louisiana (*6*), but little is known about their feeding habits in natural conditions. To evaluate the risk for Chagas disease (based on human/vector/parasite contact) and determine the feeding behavior of the species *T. sanguisuga*, we molecularly identified the blood meal sources and *T. cruzi* infection in *T. sanguisuga* kissing bugs.

## The Study

In 2007, at the site of the first autochthonous case of transmission of Chagas disease in Louisiana, 49 *T. sanguisuga* (16 male and 33 female) bugs were collected and identified (*6**–**8*). DNA was isolated from the abdomen of each bug by using the DNeasy Blood and Tissue Kit (QIAGEN, Valencia, CA, USA), and presence of *T. cruzi* was assessed by PCR (*9*). *T. cruzi* infection was found in 27 (55.1%) bugs; prevalence did not differ significantly between males (50.0%, 8/16) and females (57.6%, 19/33) (Fisher exact test; p = 0.76) (Technical Appendix Table).

Blood meals were detected by using PCR with universal vertebrate primers targeting the 12S ribosomal RNA gene (*10*). The PCR products were purified and cloned to enable detection of multiple blood sources in a single bug. For cloning, the p-GEM-T Easy Vector System (Promega, Madison, WI, USA) was used; for the ligation step, the DNA-to-vector ratio was 3:1. After transformation, up to 8 transformants per bug were randomly selected and sequenced. Blood meals were detected in 45 (92%) of 49 bugs, and 43 (96%) of the 45 detected blood meals were successfully cloned (Technical Appendix Table).

Blood meal sources were inferred by using BLAST (http://www.ncbi.nlm.nih.gov/blast/Blast.cgi); >97% identity was considered a match. From the 43 bugs, 201 vertebrate 12S sequences were obtained. In all, 8 vertebrate blood-source species were identified. Multiple blood source species were identified in 21 (48.8%) of 43 bugs; the maximum number of blood meal sources was 4 (Technical Appendix Table), confirming the ability of the cloning approach to identify multiple blood meals. The average number of blood source species detected per bug was 1.6. The predominant blood source was the American green tree frog (*Hyla cinerea*), found in 53.5% of triatomines; the second most predominant was the human (*Homo sapiens*), found in 48.8%, followed by the raccoon (*Procyon lotor*), found in nearly 30% of triatomines (Figure). Less prevalent blood sources included cow (*Bos taurus*), dog/wolf (*Canis lupus*), squirrel (*Sciurus carolinensis*), cat (*Felis domesticus*), and woodrat (*Neotoma floridana*), each found in <15% of bugs.

**Figure:**
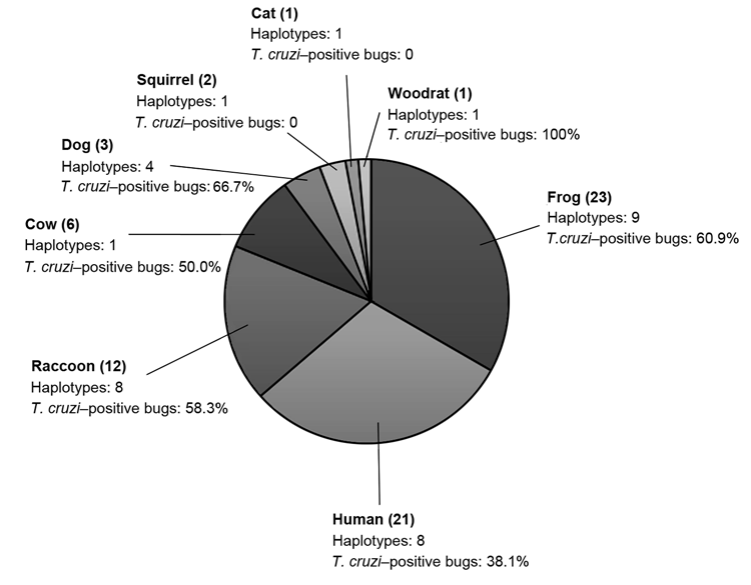
Vertebrate blood meal sources of *Triatoma sanguisuga* kissing bugs detected by 12S rDNA assay (*10*). The numbers of triatomines containing each vertebrate blood source are indicated in parenthesis. The numbers of haplotypes of each vertebrate source and the *Trypanosoma cruzi* infection prevalence in the triatomines containing this vertebrate blood source are indicated.

In total, 33 different vertebrate 12S haplotypes were found at an average of 2.1 per bug Technical Appendix Table, Figure). We found 8 human haplotypes, indicating that bugs had fed on at least 8 persons, assuming that multiple haplotypes did not result from heteroplasmy. More female than male bugs had fed on frogs (Fisher exact test, p = 0.005). Neither the average number of blood sources detected (females 1.7 ± 0.8 vs. males 1.4 ± 0.6, *t*-test; p = 0.23) nor the average number of vertebrate haplotypes found (females 2.0 ± 0.9 vs. males 2.1 ± 0.9, *t*-test; p = 0.75) differed significantly between bugs of each sex.

Of the 55% of bugs that were infected with *T. cruzi*, 61.9% had fed on frogs (incompetent *T. cruzi* host) and 38.1% on humans. We found 3 human haplotypes in *T. cruzi*–infected bugs, suggesting, in the absence of heteroplasmy, that at least 3 persons were bitten by an infected bug (Technical Appendix Table). Because only 2 persons lived at the location sampled, some bugs may have fed on visiting persons or migrated from nearby houses. No significant association was found between infection and a particular blood source, even after we removed from analysis all insects that had fed on at least 1 frog or had fed on frogs only.

## Conclusions

Our results indicate that *T. sanguisuga* kissing bugs pose an epidemiologic threat to humans and animals in Louisiana. Human/vector/*T. cruzi* contact is frequent; 55% of bugs were infected, of which nearly 40% had fed on humans.

In addition to humans, the bugs fed on a wide variety of vertebrates; multiple blood sources were detected in about half of the bugs. These observations support catholic and opportunistic feeding habits for *T. sanguisuga*, which probably feed on any available animal. The high occurrence of the American green tree frog as a blood source is not surprising because these frogs are abundant in this region (*11*). Although amphibians are incompetent *T. cruzi* hosts, frogs contribute to the epidemiology because as a blood source, they help maintain large populations of bugs near human dwellings. Further investigation could determine whether frogs also control the bug population by eating them, as do *Neotoma* spp. rats, the traditional hosts of kissing bugs in North America (*5*). Raccoons serve as sylvatic *T. cruzi* reservoirs in the southeastern United States and play an epidemiologic role because they are frequently found close to humans and, as do woodrats, they link the sylvatic cycle of the parasite with a domestic cycle (*5*). Dogs also serve as reservoirs and are at high risk for Chagas disease; many dogs in the southern United States die of this disease (*1*,*12*,*13*). In addition to the loss of companion animals, Chagas disease in animals has an evident economic effect (*13*).

Results of our study, as well as those of Stevens et al. (*10*) and Kjos et al. (*14*), reject the assertion that kissing bugs in North America prefer blood from wild animals, which has been one explanation for the low prevalence of Chagas disease in the United States. Our study provides evidence of frequent vector/human/*T. cruzi* contact in Louisiana and reveals the potential for transmission in the United States. Even if this finding does not apply to all localities (e.g., human blood has rarely been detected in triatomines from Texas [*15*]), the risk for vectorial transmission of *T. cruzi* in the United States may increase because of expansion of human settlements into formerly sylvatic areas. Moreover, the low number of Chagas disease cases reported in the United States is probably caused, at least in part, by a lack of awareness (*4*,*5*). Because knowledge of the feeding behavior of triatomines is critical for the implementation of efficient control measures, more studies of the blood sources of triatomines in North America are needed. In addition, awareness of Chagas disease and surveillance of insect vectors and human disease should be improved.

Technical Appendix Blood meal sources of *Triatoma sanguisuga* kissing bugs, determined by 12S rDNA assay, and alignment of the vertebrate 12S haplotypes detected in *T. sanguisuga* kissing bug abdomens.
