# Supplementary material for: Triatoma sanguisuga Blood Meals and Potential for Chagas Disease, Louisiana, USA
Source: Emerg Infect Dis. 2014 Dec;20(12):2141–3. doi: 10.3201/eid2012.131576 (PMC4257814; doi:10.3201/eid2012.131576)
Supplement: Technical Appendix — Blood meal sources of Triatoma sanguisuga kissing bugs, determined by 12S rDNA assay, and alignment of the vertebrate 12S haplotypes detected in T. sanguisuga kissing bug abdomens. [file 13-1576-Techapp-s1.pdf]

Article DOI: http://dx.doi.org/10.3201/eid2012.131576

# *Triatoma sanguisuga* Blood Meals and Potential for Chagas Disease, Louisiana, USA

## Technical Appendix

Technical Appendix Table. Blood meal sources of *Triatoma sanguisuga* kissing bugs, determined by 12S rDNA assay (*1*)*

| | | | | | Vertebrate blood meal source detected‡ | | | | | | | | | |
|---|---|---|---|---|---|---|---|---|---|---|---|---|---|---|
| | | | | | Taxa and haplotypes detected | | | | | | | | | |
| *T. sanguisuga* bug no. | Sex | Infection with *T. cruzi*† | Blood meal presence (vertebrate 12S amplification)† | No. clones selected and sequenced after 12S reamplification | *Hyla cinerea* (American green tree frog) | *Homo sapiens* (Human) | *Procyon lotor* (Raccoon) | *Bos taurus* (Cow) | *Canis lupus* (Dog/Wolf) | *Sciurus carolinensis* (Squirrel) | *Felis domesticus* (Cat) | *Neotoma floridana* (Eastern woodrat) | No. species detected | No. haplotypes detected |
| 1 | F | – | – | NA | | | | | | No blood meal detected | | | | |
| 2 | F | + | – | NA | | | | | | No blood meal detected | | | | |
| 3 | F | + | + | 8 | A | | | | | | | A | 2 | 2 |
| 4 | F | – | + | 7 | B | | | | | | A | | 2 | 2 |
| 5 | F | + | + | 1 | A | | | | | | | | 1 | 1 |
| 6 | F | – | + | 7 | A | A | A, B | | | | | | 3 | 4 |
| 7 | F | – | + | 1 | | B | | | | | | | 1 | 1 |
| 8 | M | + | + | 3 | B | A | | | | | | | 2 | 2 |
| 9 | F | – | + | 7 | | A | | | | | | | 1 | 1 |
| 10 | F | + | + | 7 | C | A | | | | | | | 2 | 2 |
| 11 | M | – | + | 3 | | A | | | | | | | 1 | 1 |
| 12 | M | – | + | 4 | | | A, C, D | | | | | | 1 | 3 |
| 13 | F | – | + | 6 | A | | | | | | | | 1 | 1 |
| 14 | F | + | + | 3 | D | A | | | | | | | 2 | 2 |
| 15 | F | + | + | 5 | A | | A, E | | | | | | 2 | 3 |
| 16 | M | – | + | 2 | | A, C | | | | | | | 1 | 2 |
| 17 | F | + | – | NA | | | | | | No blood meal detected | | | | |
| 18 | F | + | + | 2 | A | | | | | | | | 1 | 1 |
| 19 | F | + | + | 3 | A, E | D | | | | | | | 2 | 3 |
| 20 | M | + | + | 7 | A | | A | | | | | | 2 | 2 |
| 21 | F | + | + | 6 | A, F | | | | | | | | 1 | 2 |
| 22 | M | + | + | 6 | | | A, F | | | | | | 1 | 2 |
| 23 | F | + | + | 6 | | | A | | | | | | 1 | 1 |
| 24 | F | – | + | 8 | A | | A | | | | | | 2 | 2 |
| 25 | F | + | + | 5 | B | | A | A | A | | | | 4 | 4 |
| 26 | M | – | + | 8 | A,G | A | | | | | | | 2 | 3 |
| 27 | F | – | + | 7 | A | A, E | | | | | | | 2 | 3 |
| 28 | F | + | + | 0 | | | | | | No transformants obtained | | | | |
| 29 | F | + | + | 3 | B, H | | | | | | | | 1 | 2 |
| 30 | F | – | + | 4 | A | | A | | | | | | 2 | 2 |

| | | | | | Vertebrate blood meal source detected‡ | | | | | | | | | |
|---|---|---|---|---|---|---|---|---|---|---|---|---|---|---|
| | | | | | Taxa and haplotypes detected | | | | | | | | | |
| *T. sanguisuga* bug no. | Sex | Infection with *T. cruzi*† | Blood meal presence (vertebrate 12S amplification)† | No. clones selected and sequenced after 12S reamplification | *Hyla cinerea* (American green tree frog) | *Homo sapiens* (Human) | *Procyon lotor* (Raccoon) | *Bos taurus* (Cow) | *Canis lupus* (Dog/Wolf) | *Sciurus carolinensis* (Squirrel) | *Felis domesticus* (Cat) | *Neotoma floridana* (Eastern woodrat) | No. species detected | No. haplotypes detected |
| 31 | F | + | + | 3 | A | | | | | | | | 1 | 1 |
| 32 | F | – | + | 6 | A, I | | | | | | | | 1 | 2 |
| 33 | M | + | + | 6 | | | | | A, B, C | | | | 1 | 3 |
| 34 | F | + | + | 7 | | A | | A | | | | | 2 | 2 |
| 35 | M | – | + | 5 | | | | | A, D | | | | 1 | 2 |
| 36 | F | + | – | NA | | | | | | No blood meal detected | | | | |
| 37 | M | – | + | 2 | | A | | | | | | | 1 | 1 |
| 38 | F | + | + | 0 | | | | | | No transformants obtained | | | | |
| 39 | M | – | + | 1 | | | | | | A | | | 1 | 1 |
| 40 | F | + | + | 5 | | | A, G | | | | | | 1 | 2 |
| 41 | F | – | + | 5 | A | A | H | | | | | | 3 | 3 |
| 42 | F | – | + | 6 | | A, F | | | | | | | 1 | 2 |
| 43 | F | – | + | 8 | | A | | A | | | | | 2 | 2 |
| 44 | M | + | + | 5 | | A | | | | | | | 1 | 1 |
| 45 | M | + | + | 8 | | A, G | | A | | | | | 2 | 3 |
| 46 | M | + | + | 7 | | A | A | | | | | | 2 | 2 |
| 47 | M | – | + | 8 | | A, H | | A | | A | | | 3 | 4 |
| 48 | M | + | + | 8 | A, B | | | | | | | | 1 | 2 |
| 49 | F | – | + | 8 | | A | | A | | | | | 2 | 2 |
| **Total** | | **27/49 (55.1%)** | **45/49 (92%)** | **227** | | | | | | | | | | |
| Average | | | | 5.04 clones/bug (n = 45) | | | | | | | | | 1.6 species/ bug (n = 43) | 2.1 haplotypes/ bug (N = 43) |
| Range | | | | 0–8 clones/bug (n = 45) | | | | | | | | | 1–4 species/ bug (n = 43) | 1–4 haplotypes/ bug (n = 43) |

*NA, not applicable

†To rule out PCR inhibition, the samples for which no vertebrate or parasite DNA was detected were re-amplified after adding 100 ng of positive control DNA to the PCR mixture. For the only sample with these results (bug #1), PCR inhibition was ruled out as the added DNA was amplified.

‡Blood meal sources were inferred by using BLAST with ≥ 97% identity as the criterion for a match.

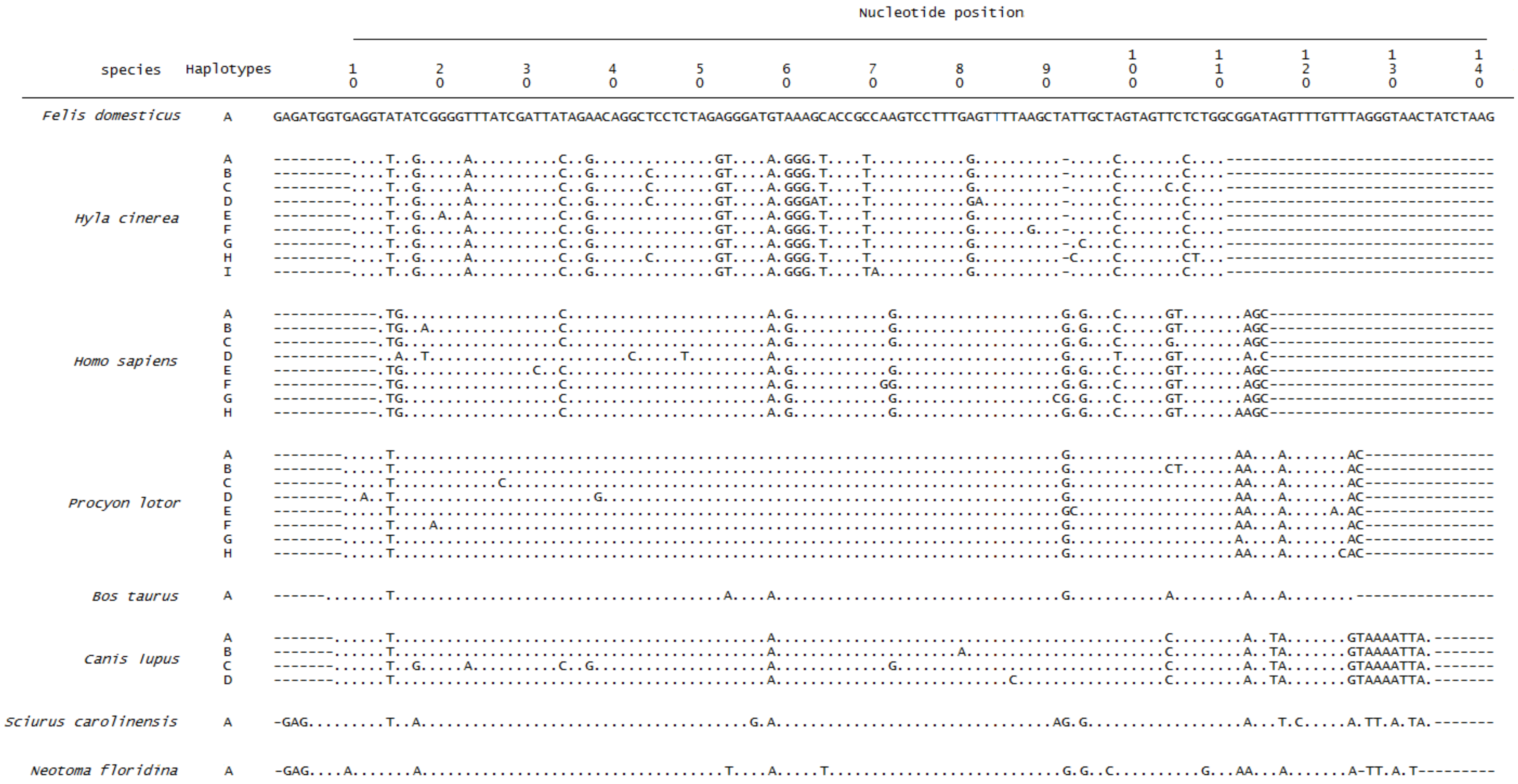


Technical Appendix Figure. Alignment of the vertebrate 12S haplotypes detected in *Triatoma sanguisuga* kissing bug abdomens. Dots indicate identity with nucleotide in first sequence. Haplotypes were defined on alignments of 100 bp (American green tree frog [*Hyla cinerea*]), 103 bp (human [*Homo sapiens*]), 118 bp (raccoon [*Procyon lotorl*]), 119 bp (cow [*Bos Taurus*]), 127 bp (dog/wolf [*Canis lupus*]), 133 bp (squirrel [*Scirus carolinensis*]), 141 bp (cat [*Felis domesticus*]), or 130 bp (woodrat [*Neotoma floridana*]). The number of haplotypes is the minimum number of specimens fed on, in the absence of heteroplasmy, because different individuals may share 12S haplotypes and only a subset of clones were sequenced.

**Reference**

1. Stevens L, Dorn PL, Hobson J, de la Rua NM, Lucero DE, Klotz JH, et al. Vector blood meals and Chagas disease transmission potential, United States. Emerg Infect Dis. 2012;18:646–9. PubMed http://dx.doi.org/10.3201/eid1804.111396
